# The Relationship Between Posttraumatic Cognitive Change, Posttraumatic Stress Disorder, and Posttraumatic Growth Among Chinese Adolescents After the Yancheng Tornado: The Mediating Effect of Rumination

**DOI:** 10.3389/fpsyg.2018.00474

**Published:** 2018-04-09

**Authors:** Yi Zhang, Wei Xu, Guangzhe Yuan, Yuanyuan An

**Affiliations:** College of Psychology, Nanjing Normal University, Nanjing, China

**Keywords:** posttraumatic cognitive change, posttraumatic stress disorder, posttraumatic growth, intrusive rumination, deliberate rumination

## Abstract

The aim of this study was to explore the different cognitive processes involved in the development of posttraumatic stress disorders (PTSDs) and posttraumatic growth (PTG). One year after the Yancheng tornado, 455 middle school students were assessed to study how posttraumatic cognitive change (PCC) influenced PTSD and PTG among traumatized Chinese adolescents through the role of rumination. The results indicated that intrusive rumination partly mediated the relationship between PCC and PTSD, and deliberate rumination partly mediated the relationship between PCC and PTSD and completely mediated the relationship between PCC and PTG. These results suggest that the cognitive processes of PTSD and PTG are different. Furthermore, the study also suggests that PTSD and PTG can coexist in individuals. This study may offer some suggestions for clinical practice after traumatic events.

## Introduction

A tornado hit Yancheng City, Jiangsu Province on June 23, 2016. This tornado claimed 99 lives and caused more than 3,800 flats to collapse (Lyu et al., 2017). Many studies have shown that individuals who experienced this form of trauma may show negative psychological reactions such as depression, anxiety, posttraumatic stress disorder (PTSD), and so on (Goenjian et al., 2000; Norris et al., 2002; Furr et al., 2010). Thus, research shows that there is a high incidence of PTSD from the impact of tornadoes. For example, after the tornado that affected Joplin, MO, United States, the incidence of PTSD in adolescents was found to be 12.63% 6 months after the event, and 26.74% 2.5 years later (Houston et al., 2015). Adams et al. (2014, 2015) reported that 6.7% of adolescents met the diagnostic criteria for PTSD, after a spring 2011 tornado in Alabama and Joplin. Some studies also show that adolescents have been reporting more psychological symptoms after traumatic events than adults (Norris et al., 2002; McMahon et al., 2003; Crews et al., 2007). Therefore, it is necessary to pay close attention to the mental health problems of Chinese adolescents after a tornado.

Researchers have gradually found that adolescents with trauma not only have negative psychological reactions but also may have positive psychological changes, such as posttraumatic growth (PTG). PTG is the experience of positive change that occurs from struggle with highly challenging life crises. It mainly includes change in self, changes in interpersonal relationships, and changes in life attitudes (Tedeschi and Calhoun, 1995, 1996). Previous studies have found that people experiencing different traumas are likely to exhibit PTG (Cryder et al., 2006; Wolchik et al., 2008). A study showed that 51.1% teenagers exhibited PTG after the Wenchuan earthquake (Jin et al., 2014). So, what influences the emergence of PTSD and PTG?

### Posttraumatic Cognitive Change (PCC)

Posttraumatic cognitive change (PCC) indicates individuals’ cognitive changes (feeling guilty, worrying about bad things, feeling permanently harmed, and going crazy) after traumatic experiences (Wang et al., 2013). Xu et al. (2017) found that PCC was positively correlated with PTSD symptoms among Chinese adolescents following a tornado.

The social cognitive theory of PTSD holds that cognition has an important role in PTSD (Horowitz, 1986; Janoff-Bulman, 1992). For example, Horowitz’s stress-response syndromes theory (Horowitz, 1973) holds that PTSD is related to the cognitive processing of trauma information and the change of existing models and beliefs. Janoff-Bulman’s cognitive-appraisal theory holds that the reason why people exhibit PTSD is that some of their basic beliefs about the world are shattered (Janoff-Bulman, 1992). However, many existing studies of cognitive behavioral therapy for PTSD also support that the adjustment of individual negative cognition can effectively improve the PTSD symptoms of individuals (Foa and Rauch, 2004; Ehlers et al., 2010; Kleim et al., 2013; Zhen et al., 2016).

In addition, some theories show that cognitive changes can also influence PTG. Tedeschi and Calhoun (1995) mentioned that cognitive processing could be suggested as an important factor in the development of PTG. The comprehensive model of PTG proposed by Calhoun and Tedeschi (2004) also proved that the psychological stress and cognitive imbalances caused by traumatic events stimulate people’s cognitive processing. If this is constructive cognitive processing, individuals will repeatedly think about themselves, others and the world, and the value of the event, which will contribute to the formation of PTG (Calhoun and Tedeschi, 2006).

A growing of empirical studies have explored the relationship between cognitive processes of individuals and PTSD or PTG. For example, a study among adolescent survivors after the Wenchuan earthquake, core belief challenge had effects on PTG (Zhou et al., 2014). A study among college students also confirmed that challenge to core beliefs was the main predictor of PTG (Lindstrom et al., 2013). However, few studies have explored the different effects of cognitive processes on both PTSD and PTG. Based on this review of previous theoretical and empirical research, this study proposes PCC as a research variable of cognitive processes which may influence the emergence of PTSD and PTG.

### Influence of Different Rumination on PTSD and PTG

The cognitive process of repeatedly thinking about traumatic events and their consequences is called rumination (Watkins, 2008). It includes two forms: intrusive rumination and deliberate rumination. The former means that traumatic events invade individual cognition in unexpected ways (Nolen-Hoeksema and Davis, 2004). The latter refers to individual actively and repeatedly thinking about traumatic events and related clues (Calhoun et al., 2000; Cann et al., 2010a). Previous studies have indicated that rumination mediates the relationship between core belief challenge and PTG (Zhou et al., 2014), and that rumination can mediate the relationship between the severity of the event and posttraumatic stress symptoms (Andrades et al., 2017). Therefore, different ways of thinking about a traumatic event may be responsible for the generation of PTSD or PTG.

Although many previous studies have found that rumination is significantly related to PTG and PTSD (Ehring and Ehlers, 2014; García et al., 2016; Zhou et al., 2016), there are different opinions about the influence of the different types of rumination on PTSD and PTG. Some researchers have suggested that intrusive rumination forces individuals to focus on traumatic events and negative thoughts and feelings, leading to negative perceptions of events and the future, which results in PTSD (Taku et al., 2009; Michl et al., 2013; Andrades et al., 2017). In another view, intrusive rumination after a traumatic event has a direct positive effect on PTG (Taku et al., 2008; Zhou et al., 2017). According to the PTG model of Calhoun and Tedeschi (2006), deliberate rumination can provide individuals with opportunities for further cognitive processing of a traumatic event. Thus, it is conducive to rebuild understanding of the world, the self and others, which results in PTG (Cann et al., 2010b). Deliberate rumination can also reduce PTSD symptoms (Brewin et al., 2000).

### The Present Study

Most of this research has, however, been focused on adult samples. Given that the cognitive ability of adolescents could still be developing, the cognitive system has great plasticity. So, it is important to focus on the level of cognitive development of adolescents. Adolescents’ cognitive resources are likely to be occupied by traumatic experiences, and their attention may be difficult to shift from negative traumatic information, which leads to more and more serious cognitive conflicts, thereby adversely affecting the generation of PTG (Chuen Yee Lo et al., 2012; Zhou et al., 2014).

The aim of this study was to examine the role of PCC in the development of PTSD and PTG, and to compare the different mediation effects of different types of rumination among adolescents after the Yancheng tornado. We proposed the following five hypotheses: (1) PCC is positively correlated with PTSD and PTG; (2) PCC is positively correlated with rumination; (3) Deliberate rumination leads directly to PTG, while intrusive rumination leads to PTSD; (4) Intrusive rumination mediates the relationships between PCC and PTSD; and (5) Deliberate rumination mediates the relationships between PCC and PTG.

## Materials and Methods

### Participants and Procedure

This study was conducted 1 year after the Yancheng tornado of June 23, 2016. Participants were 455 adolescents from two middle schools in the main area hit by the tornado (in Funing, Yancheng). Excluding invalid questionnaires from 12 participants, we were left with 443 responses. The mean age of the participants was 14.44 (*SD* = 0.714), and 208 (47%) of them were male and 235 (53%) were female. Of the respondents, 281 (63.4%) were in grade seven, 157 (35.4%) were in grade eight, and 5 (1.1%) were in grade nine.

The questionnaires were completed class by class, and we randomly selected nine classes in the schools. After the agreement of the school, the class teacher and the students themselves, participants were informed that they would be taking part in a study on distress. Students completed the pencil-and-paper questionnaires in quiet classrooms, and it took approximately 20 min to finish all the questionnaires. Upon completion of the study, the researchers conducted a 10-min group game with the participants as a reward for their participation. This study was approved by the Ethics Committee of Nanjing Normal University. The parents of the participants under the age of 16 in this study have given written informed consent.

### Measures

Posttraumatic stress disorder symptom levels were measured by the Child PTSD Symptom Scale (CPSS; Foa et al., 2001), which evaluates the Diagnostic and Statistical Manual of Mental Disorders-IV (DSM-IV) PTSD symptoms on occurrence and frequency. The Chinese version of CPSS was revised by Zhang (2009). This is a 17-item self-report scale. Adolescents need to report the presence and frequency of their symptoms during the previous 2 weeks on a four-point Likert scale ranging from 0 (not at all) to 3 (almost always). The CPSS includes three subscales, namely intrusion, avoidance, and hyper-arousal. Higher scores indicate greater severity of PTSD symptoms. In the current study, the Cronbach’s α for the subscales—intrusion, avoidance, and hyper-arousal—are 0.77, 0.83, 0.74, and 0.78, respectively. The Cronbach’s α for the CPSS is 0.89.

The Posttraumatic Growth Inventory (PTGI) was developed by Tedeschi and Calhoun (1996), which measures positive changes after suffering from traumatic events. In this study, PTG was measured using the revised Chinese version of the Post-Traumatic Growth Inventory (PTGI-R; Wang et al., 2011; Zhou et al., 2014). This version consists of 22 items, and it is a six-point scale ranging from 0 (I did not experience this change after the traumatic event) to 5 (I experienced this change to a very great degree after the traumatic event). Three factors were observed: changes in self-perception, changes in interpersonal relationships, and changes in philosophy of life. Higher scores indicate more PTG traumatic events. In the current study, the Cronbach’s α for the subscales—changes in self-perception, changes in interpersonal relationships, and changes in philosophy of life—are 0.92, 0.88, 0.74, and 0.80, respectively. The Cronbach’s α for the PTGI is 0.96.

Posttraumatic cognitive change is used to assess the negative cognitive changes after traumatic events (Wang et al., 2013). Items are rated on a five-point scale ranging from 0 (not at all) to 4 (very much so). Sample items include “I now believe that the world is a very dangerous place,” and “I am worried about bad things happening in the future.” Higher scores indicate more negative cognition changes. The Chinese version of PCC was revised by Xu et al. (2014). In this study, the internal reliability of the questionnaire was good (α = 0.80).

The original Event-Related Rumination Inventory was developed by (Cann et al., 2011), which measures rumination. The Event-Related Rumination Inventory modified by Zhou et al. (2014) was used in this study. It consists of 20 items and is divided into two dimensions: intrusive rumination and deliberate rumination. The items are rated on a six-point scale that ranges from 0 (not at all) to 5 (always). The Cronbach’s alpha in this study was 0.92.

### Data Analysis

The descriptive statistics, Pearson correlation analysis, and analysis of variance used in this study were provided by SPSS 22.0. We conducted an analysis of missing data in variables and found that missing data across all items were less than 2.5%. Little’s Missing Completely at Random test suggested that the rate of missing data was equivalent across all measures (*p* > 0.05). Missing data was handled by using full information maximum likelihood estimates (FIML) in the models. AMOS 24.0 was adopted to test the hypothesized model and mediation effects. Model fit was evaluated based on the Chi square statistic (χ^2^), as well as with the goodness-of-fit index (CFI), the Tucker–Lewis index (TLI), the root mean square error of approximation (RMSEA), and the standardized root mean residual (SRMR). According to the recommendations of Hu and Bentler (1999), A model is typically accepted as an adequate fit when RMSEA and SRMR < 0.08, CFI and TLI > 0.90. To evaluate the mediations, a corrected-bias bootstrap estimation was performed with 1,000 samples bootstrap, and a 95% confidence interval. In this case, mediation exists if the zero is not included in the interval confidence (MacKinnon et al., 2004).

To examine common method bias, we used Harman’s single-factor test (Podsakoff et al., 2003). All items relevant to the study were subjected to an exploratory factor analysis. The results show that 11 factors can be obtained, while no single factor accounts for a majority of the covariance among the variables. Therefore, no significant common method bias existed in the current study.

## Results

### Descriptive Statistics and Correlations Among Main Measures

Descriptive statistics and Pearson correlations are shown in **Table 1**. There are significant correlations between all the main variables except for the relationship between PTG and PTSD; PTG and intrusive rumination.

**Table 1:** Means (M), standard deviations (SD), and correlations of all the variables.

	*M ± SD*	1	2	3	4	5
1. PCC	4.80 ± 4.11	1				
2. Intrusive rumination	7.92 ± 7.21	0.43^∗∗∗^	1			
3. Deliberate rumination	10.10 ± 7.15	0.31^∗∗∗^	0.63^∗∗∗^	1		
4. PTSD	11.15 ± 8.11	0.60^∗∗∗^	0.48^∗∗∗^	0.31^∗∗∗^	1	
5. PTG	51.72 ± 24.40	0.10^∗^	0.08	0.27^∗∗∗^	0.02	1

*PCC, posttraumatic cognitive change; PTSD, posttraumatic stress disorder; PTG, posttraumatic growth, ^∗^p < 0.05, ^∗∗∗^p < 0.001.*

### Structural Equation Model Analyses

#### Measurement Model Results

**FIGURE 1:**
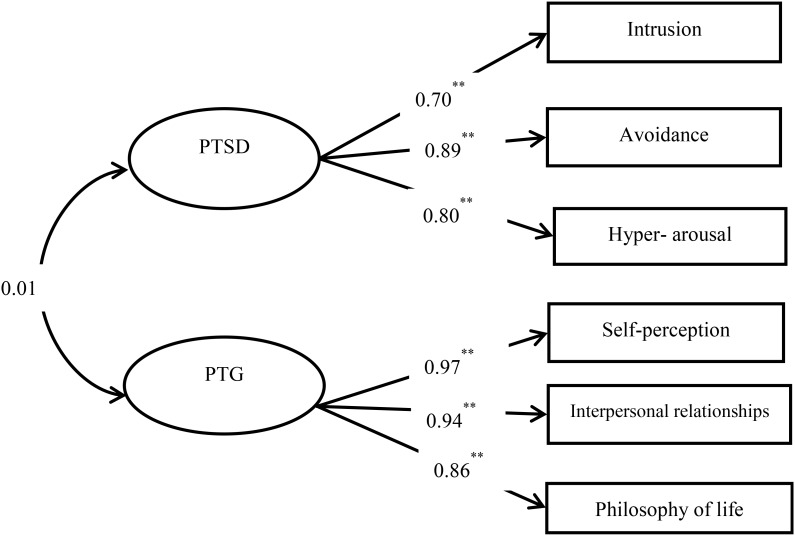
The measurement model of two latent variables: posttraumatic stress disorder (PTSD) and posttraumatic growth (PTG). All the coefficients are standardized estimates, ^∗∗^*p* < 0.01.

We first built a measurement model that included two latent variables: PTSD and PTG. The PTSD latent variable was evaluated using scores for the CPSS subscales of intrusion, avoidance, and hyper-arousal (Foa et al., 2001), whereas the PTG latent variable was evaluated using changes in self-perception, changes in interpersonal relationships, and changes in philosophy of life (Zhou et al., 2014). In this measurement model (**Figure 1**), correlations were specified between PTSD and PTG. Factor loadings of the manifest indicators on their respective latent variables were estimated freely. The measurement model presented satisfactory fit indices: *χ*^2^ = 12.756, *df* = 8, *χ*^2^/*df* = 1.594, CFI = 0.997, TLI = 0.995, RMSEA = 0.037. According to the recommendations of Hu and Bentler (1999), CFI values ≥0.90, TLI values ≥0.90, and RMSEA values ≤0.08 are all considered adequate and indicative of good fit. These results indicated that the measurement model was sound, and that it was appropriate to conduct further analysis of the SEM.

#### Structural Model Results

**FIGURE 2:**
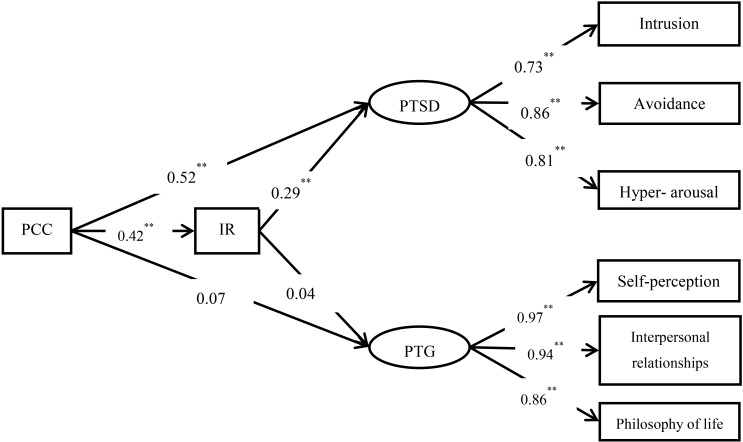
The pathways from posttraumatic cognitive change (PCC) to posttraumatic stress disorder and posttraumatic growth (PTG) through intrusive rumination. All the coefficients are standardized estimates (model 1), ^∗∗^*p* < 0.01.

**FIGURE 3:**
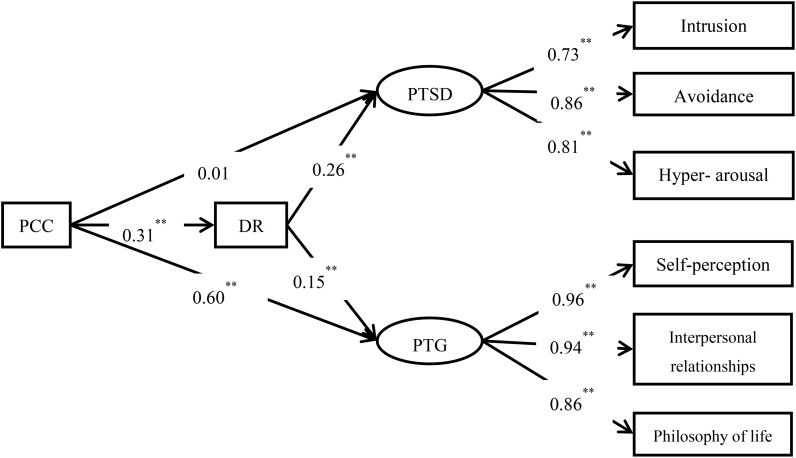
The pathways from posttraumatic cognitive change (PCC) to posttraumatic stress disorder and posttraumatic growth (PTG) through deliberate rumination. All the coefficients are standardized estimates (model 2), ^∗∗^*p* < 0.01.

The hypothesized model 1 and model 2 mentioned above were evaluated (**Figures 2**, **3**). Results showed that model 1 had a good fit to the data: *χ*^2^ = 39.110, *df* = 17, *χ*^2^*/df* = 2.301, CFI = 0.990, TLI = 0.983, RMSEA = 0.054; and that model 2 also fit the data well: *χ*^2^= 43.288, *df* = 17, *χ*^2^*/df* = 2.546, CFI = 0.988, TLI = 0.980, RMSEA = 0.059. **Figures 2**, **3** show the path coefficients of the models.

To evaluate the significance levels of the indirect effects in the models, we conducted bias-corrected bootstrap tests with a 95% confidence interval to determine the significance of the indirect effects. **Table 2** shows that intrusive rumination partly mediated the relationship between PCC and PTSD, but not the relationship between PCC and PTG. **Table 3** reveals that deliberate rumination partly mediated the relationship between PCC and PTSD and completely mediated the relationship between PCC and PTG.

**Table 2:** Bias-corrected Bootstrap Tests of the Mediating Effects (Model 1).

Paths	*β*	95% CI	
		Low	High
**Direct paths**			
PCC-PTSD	0.52^∗∗^	0.44	0.60
PCC-PTG	0.07	-0.04	0.17
**Indirect paths**			
PCC-IR-PTSD	0.12^∗∗^	0.08	0.17
PCC-IR-PTG	0.02	-0.03	0.07

*PCC, posttraumatic cognitive change; PTSD, posttraumatic stress disorder; PTG, posttraumatic growth; IR, intrusive rumination, ^∗∗^p < 0.01.*

**Table 3:** Bias-corrected Bootstrap Tests of the Mediating Effects (Model 2).

Paths	*β*	95% CI	
		Low	High
**Direct paths**			
PCC-PTSD	0.60^∗∗^	0.50	0.69
PCC-PTG	0.01	-0.10	0.11
**Indirect paths**			
PCC-DR-PTSD	0.05^∗∗^	0.02	0.08
PCC-DR-PTG	0.08^∗∗^	0.04	0.14

*PCC, posttraumatic cognitive change; PTSD, posttraumatic stress disorder; PTG, posttraumatic growth; DR, deliberate rumination, ^∗∗^p < 0.01.*

## Discussion

In this study, we assessed models to explore the relationships between PCC, intrusive rumination, deliberate rumination, and their influence on PTSD and PTG in adolescents, as affected by the tornado in Yancheng. As predicted, PCC had a positive association with PTSD and PTG. This result is consistent with previous research (Ehlers and Clark, 2000; Ehlers et al., 2010; Xu et al., 2014).

The hypothesized model fits the data well, indicating that PCC can influence PTSD and PTG through the role of rumination, however, the pathways to generate PTSD and PTG are different. This finding is supported by previous studies (Chan et al., 2011; Triplett et al., 2012). Further analyses showed that both intrusive rumination and deliberate rumination significantly mediate the relationship between PCC and PTSD.

The current study shows that PCC is positively correlated with rumination. This is understandable because individuals can exhibit negative cognitive changes after traumatic events. For instance, they may have such thoughts: “I now believe that the world is a very dangerous place,” and “I have been permanently harmed (not considering any physical injuries sustained) by the event (Wang et al., 2013).” This can lead to individual psychological discomfort, which forces individuals to immerse themselves in the traumatic events, and repeatedly think on the traumatic events. This kind of compulsive brooding is intrusive rumination. At the same time, it may also make individuals actively aware of the undesirability of negative cognition, and assist them to adjust their cognition of the event. This kind of active reflective pondering is deliberate rumination. Intrusive rumination after a traumatic event forces an individual to focus on traumatic events, which may increase individuals’ negative thoughts and emotion about the consequences of those events. Thus, intrusive rumination was positively correlated with PTSD. This result shows coherence with the findings from previous research (Taku et al., 2009; Michl et al., 2013; Roley et al., 2015). In addition, we observed that deliberate rumination was also positively correlated with PTSD. This result is inconsistent with previous studies (Cann et al., 2011; Andrades et al., 2017), which may be due to the fact that deliberate rumination makes adolescents overly exposed to traumatic experiences, and these may cause negative emotional reactions resulting in PTSD. Another explanation for this finding could be that repeatedly thinking about traumatic events may cause the cognitive resources of adolescents to be occupied by such traumatic events. As a result, they may unduly focus on traumatic events and their negative effects, which may result in PTSD (Watkins and Brown, 2002; Chuen Yee Lo et al., 2012).

In turn, the relationship between PCC and PTSD can be explained by the social cognitive theory of PTSD (Horowitz, 1973). This result is consistent with previous research (Ehlers and Clark, 2000; Papageorgiou and Wells, 2003; Ehlers et al., 2010; Xu et al., 2014). Individuals generally have stable cognitive models and beliefs about the world in their minds. After traumatic events, cognitive processing of trauma information is performed to incorporate new cognitive models. The information may also be shown as flashbacks or nightmares. Thus, cognitive changes can positively lead to PTSD through intrusive or deliberate rumination. This means that adolescents experience more negative cognitive changes after an event such as the tornado we studied, and more severe posttraumatic stress symptoms.

The other finding of this study was that PCC can predict PTG through the mediating role of deliberate rumination. This result is also consistent with previous research (Taku et al., 2009; Lindstrom et al., 2013; Andrades et al., 2017). The relationship between deliberate rumination and PTG coincides with both the theory (Calhoun and Tedeschi, 2006), and with empirical research (Cann et al., 2010b; Kilmer et al., 2014; Taku et al., 2015; Wu et al., 2015). Deliberate rumination means that individuals actively and repeatedly think about the meaning and value of a traumatic event, and consequently reconstruct their cognition of life and the world, which may facilitate the development of PTG. In addition, the results of the relationship between PCC and PTG support Janoff-Bulman’s (2006) shattered world assumption, and Calhoun and Tedeschi’s (2006) model of PTG. When individuals experience a traumatic event, their original assumptions and beliefs about the world are challenged, and then PCCs have taken place. Such cognitive changes will force individuals to think repeatedly to find out the meaning of the event, and thus reconstruct their cognition of the world and self. The positive and constructive version of cognitive processing is deliberate rumination, which results in PTG.

In general, this study proved that individuals’ negative cognitive changes after traumatic events may lead to two types of rumination. The different types of rumination are an important factor in determining what kind of posttraumatic psychological reactions (PTSD and PTG) will develop in the end. When negative cognition changes produce deliberate rumination which is active and adaptive, this is conducive to the production of PTG. Otherwise, when negative cognition changes produce intrusive ruminations that are forced and non-adaptive, this may result in PTSD. The study also supports the hypothesis proposed by Chan et al. (2011), that there are different pathways for PTSD and PTG, indicating that PTSD and PTG are two different post-traumatic outcomes.

This study has some limitations. First, its design is cross-sectional, thus to explore the causality of these variables, further modeling should be corroborated by longitudinal or intervention studies. Second, the participants in this study are adolescents who experienced the Yancheng tornado in China, thus generalizations to other groups of people with different traumatic experiences must be made with caution. Third, while this study proposed rumination as the mediation variable to explain PTSD and PTG, there may be other mediation variables that can explain the different processes between PTSD and PTG.

Despite these limitations, this study contributes to our understanding of the differences of cognitive processes that lead to PTSD and PTG. Furthermore, it is important to find the cognitive processes that lead to PTG in adolescents affected by traumatic events. From this perspective, this study may offer some suggestions for clinical practice after traumatic events. It is important to consider the level of cognitive development of adolescents. Simultaneously, clinical intervention should also pay attention to measures designed to alleviate the intrusive rumination of adolescents after traumatic events, to reduce the incidence of PTSD.

## Author Contributions

YZ designed the study and wrote the manuscript. WX analyzed the data. YA and GY modified the manuscript. All authors have seen and approved the final version of the manuscript before submission.

## Conflict of Interest Statement

The authors declare that the research was conducted in the absence of any commercial or financial relationships that could be construed as a potential conflict of interest.
